# Pharmacological Inhibition of FKBP51 Mitigates Early Life Adversity‐Induced Social Deficits in Male Mice

**DOI:** 10.1002/advs.76040

**Published:** 2026-06-09

**Authors:** Joeri Bordes, Xiuqi Ji, Serena Gasperoni, Choham Sudre‐Chinsky, Amirali Kalbasi, Daniela Harbich, Cornelia Flachskamm, Paula Fontanet, Sowmya Narayan, Manfred Uhr, Christian Namendorf, Camilla Bellone, Alon Chen, Felix Hausch, Juan Pablo Lopez, Mathias V. Schmidt

**Affiliations:** ^1^ Research Group Neurobiology of Stress Resilience Max Planck Institute of Psychiatry Munich Germany; ^2^ Department of Basic Neuroscience University of Geneva Geneva Switzerland; ^3^ Department of Neuroscience Karolinska Institutet Stockholm Sweden; ^4^ Core Unit Analytics and Mass Spectrometry Max Planck Institute of Psychiatry Munich Germany; ^5^ Department of Brain Science Weizmann Institute of Science Rehovot Israel; ^6^ Department of Chemistry and Biochemistry Clemens‐Schöpf‐Institute Technical University Darmstadt Darmstadt Germany; ^7^ Center For Synthetic Biology Technical University Darmstadt Darmstadt Germany; ^8^ Institute of Pharmaceutical and Biomedical Science (IPBS) Johannes Gutenberg University Mainz Germany

**Keywords:** fkbp5, glucocorticoid, glucocorticoid receptor, mediator, neuroscience, nucleus accumbens, prefrontal cortex, psychopathology, social defeat, transcriptome

## Abstract

Early life adversity (ELA) is a major risk factor for psychiatric disorders, but targeted preventative strategies are lacking due to poor mechanistic insight. The FKBP51 protein, a co‐chaperone of the glucocorticoid receptor, is a key mediator of stress vulnerability. We tested if pharmacological inhibition of FKBP51 with the selective inhibitor SAFit2 prevents the long‐term consequences of ELA. Male mice exposed to ELA exhibited persistent deficits in social behavior, manifesting as social subordination in adolescence and adulthood. Early‐life SAFit2 treatment fully rescued these ELA‐induced behavioral impairments. Transcriptional profiling across six stress‐relevant brain regions revealed that SAFit2 normalized ELA‐driven gene expression changes, particularly in the medial prefrontal cortex and nucleus accumbens. Functional analysis showed the rescue converged on immunoregulatory and neuroactive ligand–receptor signaling pathways. Our findings establish FKBP51 as a critical pharmacological target for reversing the lasting impact of early life adversity on brain function, offering a path toward preventative treatment for ELA‐related psychopathology.

## Graphical abstract

Early life adversity triggers persistent social subordination and brain‐wide molecular dysregulation. Pharmacological inhibition of the stress‐mediator FKBP51 during the adversity period prevents these long‐term deficits and restores normative social hierarchy. This rescue correlates with normalized transcriptional signatures in the prefrontal cortex and nucleus accumbens, positioning FKBP51 as a promising preventative target for stress‐related psychopathology.

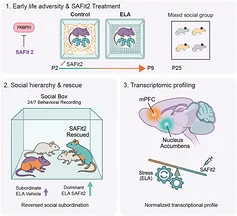

## 1 Introduction

Early life adversity (ELA), including childhood trauma and neglect, is a significant societal problem with profound and lasting consequences for mental health. Epidemiological studies consistently show that ELA is one of the most powerful risk factors associated with the development of major psychiatric disorders, including Major Depressive Disorder (MDD), anxiety, and Post‐Traumatic Stress Disorder (PTSD), among others [1, 2, 3]. While a direct association is clear, the underlying molecular and neurobiological mechanisms by which early adversity becomes biologically embedded and contributes to lifelong vulnerability remain poorly understood. This lack of mechanistic insight has hampered the development of targeted, preventative interventions that could be implemented during or shortly after periods of early trauma.

A growing body of evidence highlights the FK506‐binding protein 5 (FKBP5) gene as a key mediator of gene‐environment interactions underlying the long‐term impact of ELA on psychiatric vulnerability. Notably, the rs1360780 polymorphism in the FKBP5 gene has been consistently associated with an increased risk for MDD, PTSD, and other stress‐related disorders, especially among individuals exposed to childhood trauma [4, 5, 6, 7, 8, 9]. The protein encoded by this gene, FKBP51, is a key co‐chaperone of the glucocorticoid receptor (GR), modulating its sensitivity to stress hormones. This interaction reduces GR sensitivity to glucocorticoids, weakening the negative feedback regulation of the hypothalamic‐pituitary‐adrenal (HPA) axis [10, 11]. The resulting dysregulation of the HPA axis, characterized by prolonged elevation of stress hormones, is a hallmark of many stress‐related psychiatric disorders. Supporting this, post‐mortem studies have shown increased FKBP51 expression in the brains of individuals with MDD and PTSD, indicating long‐lasting molecular alterations [12, 13]. In parallel, Fkbp5 knockout mouse models reveal a strong causal role for this gene in orchestrating individual differences in stress resilience and susceptibility, reinforcing its relevance in the pathophysiology of stress‐related disorders [14, 15, 16, 17, 18]. Recent preclinical studies have begun to map the brain region‐specific transcriptional consequences of ELA, revealing how early adversity becomes biologically embedded through persistent gene expression changes across key circuits. For example, our prior work demonstrated that ELA disrupts social hierarchy and induces cell‐type‐specific transcriptomic alterations in the ventral hippocampus, contributing to excitation/inhibition imbalance and behavioral dysfunction [19]. This expanding knowledge base offers a roadmap for identifying molecular targets and tracking the reversal of ELA‐induced pathology. Furthermore, it has been shown that Fkbp5 is already expressed in the early postnatal brain and is sensitive to early life adversity. Specifically, our recent work demonstrated that Fkbp5 is robustly expressed at P09 in regions such as the basolateral amygdala and dorsal hippocampus, and that ELA significantly increases Fkbp5 mRNA levels in the dorsal hippocampal CA1 region in males exclusively [20], indicating dynamic regulation of Fkbp5 during this critical developmental period. These findings suggest that FKBP51 is present and modifiable at the time of ELA exposure.

The convergence of genetic, molecular, and behavioral evidence positions FKBP51 as a compelling therapeutic target for mitigating the long‐lasting impact of early trauma. Until recently, a key limitation has been the absence of a selective, brain‐penetrant small molecule capable of pharmacologically inhibiting FKBP51 in vivo. The development of SAFit2, a highly selective and potent FKBP51 inhibitor, has provided a critical pharmacological tool to directly evaluate the therapeutic potential of FKBP51 inhibition [21, 22, 23, 24, 25]. SAFit2 exhibits excellent brain permeability and effectively modulates GR function in preclinical models, opening new avenues for therapeutic exploration.

Alongside these molecular insights, behavioral neuroscience has made significant strides, particularly in the domain of social behavior. Social deficits are a hallmark of numerous psychiatric disorders and represent a persistent consequence of ELA. While conventional behavioral assays have provided fundamental insights, recent advances in deep behavioral phenotyping and computer vision‐based tracking now allow for high‐resolution, unbiased quantification of complex social interactions in vivo [26, 27, 28, 29]. These sophisticated approaches deconstruct global social impairments into discrete, quantifiable components, offering a detailed view of how ELA reshapes social behavior and enabling more sensitive evaluation for the efficacy of therapeutic interventions [30].

In this study, we leveraged the selective FKBP51 inhibitor SAFit2 to test whether pharmacological intervention can abolish the long‐term neurobehavioral and transcriptional consequences of ELA. Male mice were exposed to a well‐established model of ELA and treated with either SAFit2 or vehicle control. Through automated deep behavioral phenotyping, we demonstrate that SAFit2 treatment successfully rescues ELA‐induced deficits in social behavior, in both adolescence and young adulthood. Furthermore, we performed bulk RNA sequencing across six stress‐relevant brain regions (medial prefrontal cortex; mPFC, anterior cingulate cortex; ACC, Nucleus Accumbens Core; NAcc, Basolateral amygdala (BLA), dorsal medial thalamus; DMT, and ventral hippocampus; vHipp), thereby enabling a brain‐wide screen of ELA‐ and SAFit2‐induced transcriptional alterations and demonstrating that SAFit2 normalizes these changes in a region‐specific manner. These data uncover a complex regulatory landscape, with some genes showing cross‐regional rescue, while others exhibiting region‐specific responses to SAFit2. Together, our findings support FKBP51 as a promising therapeutic target for reversing the lasting impact of early life adversity and shed light on the transcriptional mechanisms that mediate this rescue.

## 2 Results

### 2.1 The Role of SAFit2 in Shaping Physiological Hallmarks of Early Life Adversity

**FIGURE 1:**
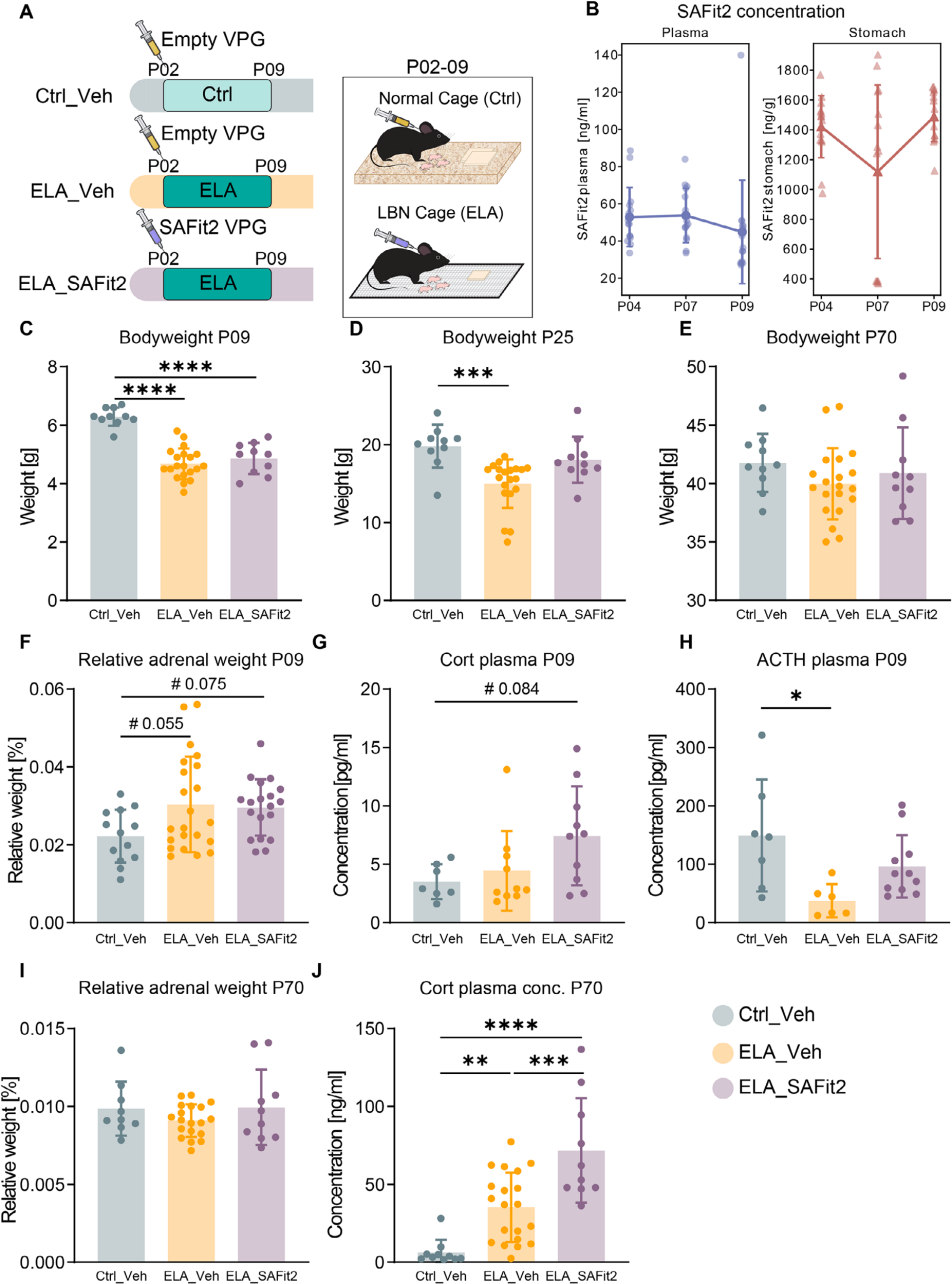
The role of SAFit2 in shaping physiological hallmarks of early life stress. (A) Experimental timeline of the nonstressed and stressed conditions with the pharmacological intervention using SAFit2. (B) The SAFit2 concentration in blood plasma and stomach in the pups during stress exposure. (C) Body weight at P09. One‐way ANOVA showed a main effect, F (2,37) = 38.49, *p* < 0.0001. Follow‐up Holm‐Šídák tests showed lower body weight in both ELA groups vs. Ctrl_veh (p‐values<0.0001). (D) Body weight at P25. Kruskal‐Wallis test showed a main effect, H (2) = 16.31, p = 0.0003. Follow‐up Dunn's tests showed lower body weight in ELA_Veh vs. Ctrl_veh (p = 0.0002), but not in ELA_SAFit2 vs. Ctrl_veh (p = 0.3247). (E) Adult weight. One‐way ANOVA showed no effect, F (2,37) = 1.099, p = 0.3439. (F) Relative adrenal weight at P09. One‐way ANOVA showed a main effect, F(2,51) = 3.279, p = 0.0457. Follow‐up Holm‐Šídák tests detected no pairwise differences for ELA_Veh vs. ELA_SAFit2: p = 0.7881, although a trend was observed for Ctrl_veh vs. ELA_Veh: p = 0.0550 and Ctrl_veh vs. ELA_SAFit2: p = 0.0753. (G) Corticosterone plasma levels at P09. One‐way ANOVA showed a trend toward a group effect, F(2,24) = 3.208, p = 0.0583. Follow‐up Holm‐Šídák tests indicated a trend for Ctrl_veh vs. ELA_SAFit2 (p = 0.0844), but not for Ctrl_veh vs. ELA_Veh (p = 0.5870) or ELA_Veh vs. ELA_SAFit2 (p = 0.1212). (H) ACTH plasma levels at P09. One‐way ANOVA showed a main effect, F(2,21) = 4.829, p = 0.0188. Follow‐up Holm–Šídák tests showed higher levels in Ctrl_veh vs. ELA_Veh (p = 0.0159), with no differences for Ctrl_veh vs. ELA_SAFit2 (p = 0.1673) or ELA_Veh vs. ELA_SAFit2 (p = 0.1673). (I) Relative adrenal weight in adulthood. One‐way ANOVA showed no effect, F(2,35) = 1.155, p = 0.3268. (J) Baseline corticosterone plasma levels in adulthood. One‐way ANOVA showed a main effect, F(2,37) = 19.81, *p* < 0.0001. Follow‐up Holm–Šídák tests showed higher levels in both ELA groups vs. Ctrl_veh (Ctrl_veh vs. ELA_Veh: p = 0.0027; Ctrl_veh vs. ELA_SAFit2: *p* < 0.0001) and in ELA_SAFit2 vs. ELA_Veh (p = 0.0005). Bar graphs and timeline are presented as the mean ± standard deviation (SD). Sample size: panel B: n = 15 for P04, n = 15 for P07, n = 15 for P09. Panels C‐E, I‐J: n = 10 for Ctrl_veh (1 excluded in panel I), n = 20 for ELA_Veh (1 excluded in panel I), n = 10 for ELA_SAFits2. Panel F: n = 13 for Ctrl_veh, n = 22 for ELA_Veh, n = 19 for ELA_SAFit2. Panel G: n = 7 for Ctrl_veh, n = 10 for ELA_Veh, n = 10 for ELA_SAFit2. Panel H: n = 7 for Ctrl_veh, n = 6 for ELA_Veh, n = 11 for ELA_SAFit2.

To determine whether SAFit2 modulates the physiological effects of early life adversity (ELA), dams received a chronic injection of SAFit2 or vehicle starting at postnatal day 2 (P02), coinciding with the onset of the ELA paradigm (Figure 1A). Across figures, different behavioral cohorts were used that are listed in Table S1. This design enabled comparison of baseline conditions (Ctrl_Veh), potential stress effects in the absence of SAFit2 (ELA_Veh), and the ability of SAFit2 to counteract ELA‐induced alterations (ELA_SAFit2). The transfer of SAFit2 to the offspring was verified by detecting stable concentrations of the compound in pup plasma and stomach contents at P04, P07, and P09 (Figure 1B).

A well‐established consequence of limited bedding and nesting (LBN) exposure is reduced body weight at P09 [20, 31]. In the present study, body weight was significantly reduced at P09 after ELA, independent of SAFit2 or vehicle treatment, confirming successful induction of chronic stress that was not prevented by SAFit2 at this early stage (Figure 1C). At P25, the stress‐induced reduction in body weight persisted in ELA vehicle animals but not in ELA_SAFit2 animals, indicating a faster recovery to baseline body weight (Figure 1D). By adulthood (P70), no differences in body weight were detected among Ctrl_Veh, ELA_Veh, and ELA_SAFit2 groups, demonstrating complete recovery in both stressed groups (Figure 1E).

Next, classical hallmarks of HPA‐axis dysregulation caused by chronic stress exposure were investigated via analysis of the relative adrenal weight, adrenocorticotropic hormone levels (ACTH), and corticosterone levels directly after ELA (P09) and into young adulthood (P70). The relative adrenal weight at P09 did not show a significant difference across conditions, although both the ELA groups showed a trend toward higher adrenal weight relative to Ctrl_Veh (Figure 1F). Furthermore, corticosterone levels at P09 showed a trend toward higher levels in ELA_SAFit2 vs. Ctrl_Veh (Figure 1G), while ACTH levels at P09 were significantly higher in Ctrl_Veh vs. ELA_Veh, with no differences between Ctrl_Veh and ELA_SAFit2 or between ELA_Veh and ELA_SAFit2 (Figure 1H), indicating condition‐specific regulation of the HPA axis in response to early life adversity and SAFit2 treatment. In adulthood, relative adrenal weight did not differ among conditions (Figure 1I). However, adult baseline corticosterone showed significantly higher levels in both ELA groups vs. Ctrl_Veh, and also significantly elevated levels in ELA_SAFit2 vs. ELA_Veh.

### 2.2 ELA‐Induced Changes in Social Behaviors are Persistent and Rescued by SAFit2

**FIGURE 2:**
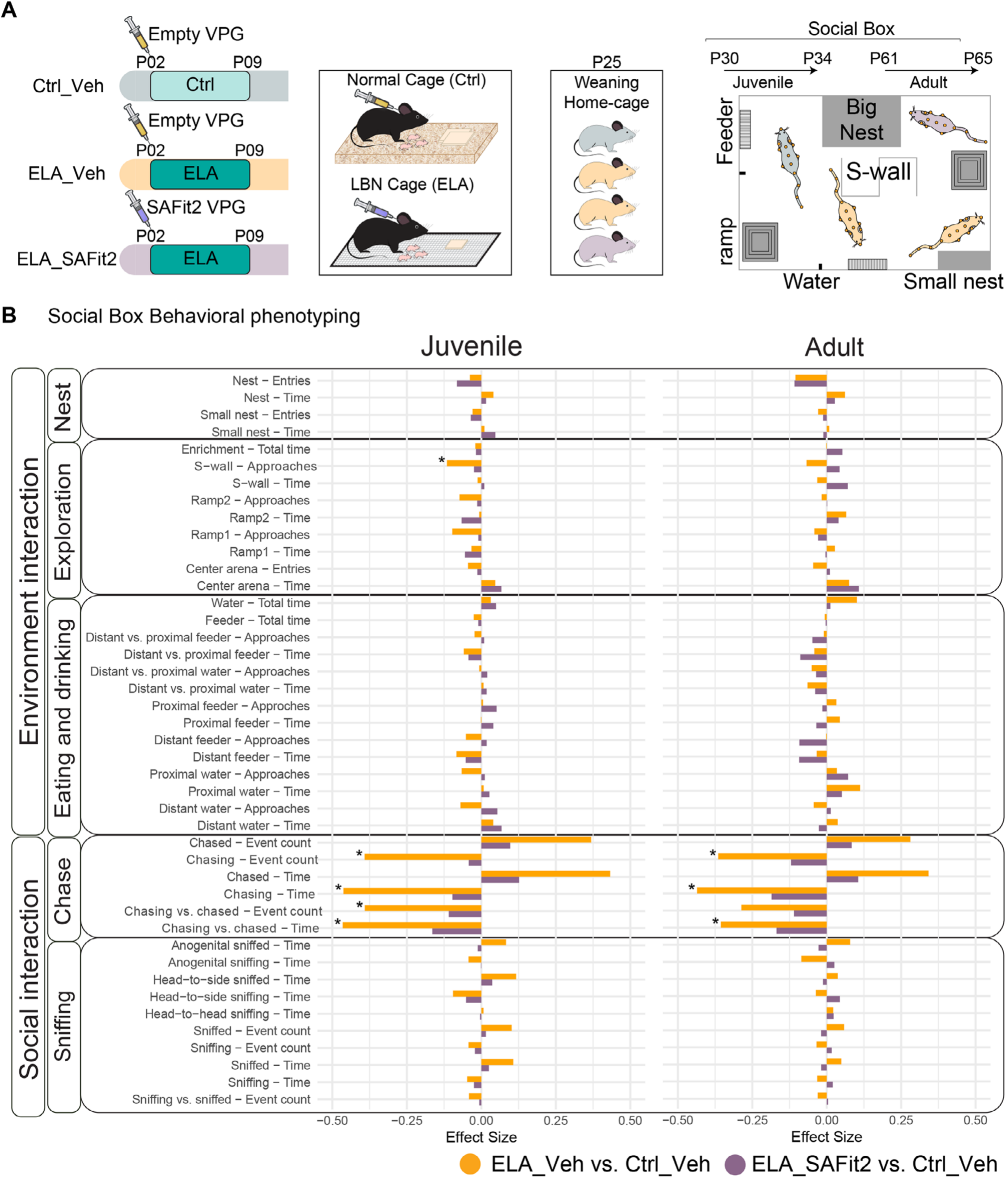
SAFit2 rescues ELA‐induced changes in social behaviors. (A) Schematic representation of the experimental timeline from birth to behavioral testing showing the three conditions: control treated with vehicle (Ctrl_Veh), ELA exposed treated with vehicle (ELA_Veh), and ELA exposed treated with SAFit2 (ELA_SAFit2). Social behavior was assessed during adolescence (P30) and early adulthood (P61) using the Social Box (SB), an enriched semi‐naturalistic environment enabling continuous behavioral tracking. (B) Summary of behaviors at P30 and P61, comparing ELA_Veh vs. Ctrl_Veh and ELA_SAFit2 vs. Ctrl_Veh using a linear mixed‐effect model. FDR adjustment was applied on p‐values. At the juvenile stage, ELA_Veh showed significantly lower total chasing time (*β = −0.463, SE = 0.177, p = 0.032*), event counts (*β = −0.392, SE = 0.156, p = 0.040*), the chase‐to‐being‐chased ratio in time (*β = −0.465, SE = 0.163, p = 0.019*) and event count (*β = −0.391, SE = 0.143, p = 0.025*), and the number of approaches to the s‐wall (*β = −0.115, SE = 0.047, p = 0.045*); in adulthood, ELA_Veh showed significantly reduced chasing time (*β = −0.435, SE = 0.145, p = 0.011*), event counts (*β = −0.364, SE = 0.125, p = 0.014*), and the chase‐to‐being chased ratio in time (*β = −0.356, SE = 0.137, p = 0.029*). Sample size: n = 10 for Ctrl_veh, n = 20 for ELA_Veh, n = 10 for ELA_SAFit2.

To investigate the long‐term impact of ELA and the potential rescuing effect of SAFit2 intervention on social behaviors, we formed ten mix‐condition groups, each containing four male mice, one Ctrl_Veh, two ELA_Veh, and one ELA_SAFit2 animal. Males were specifically selected due to the previously reported stronger ELA‐induced effects on social behavior [19]. These groups were assessed using the social box (SB) system, a semi‐naturalistic, enriched living environment that enables automated, continuous tracking of freely interacting animals. Behavioral recordings were conducted during two key developmental stages: adolescence (postnatal day 30, P30) and young adulthood (P61) (Figure 2A). The SB setup allowed for high‐resolution, longitudinal analysis of complex social interactions within a group context.

We obtained a total of 43 ethologically relevant behavioral readouts across four days (comprising four active and three inactive phases) during both juvenile (P30) and adult (P61) stages (Table S2). The behaviors were categorized into environmental interactions (nest, exploration, eating, and drinking) and social interactions (chasing, sniffing) (Figure 2B). Linear mixed‐effects modeling revealed that ELA_Veh animals exhibited significant reductions in chase‐related behaviors at both developmental stages compared to Ctrl_Veh animals (Figure 2B). At the juvenile stage, total chasing time and event counts, the chase‐to‐being‐chased ratio, and the number of approaches to the s‐wall were all significantly lower in ELA_Veh (Figure 2B). Notably, the difference in s‐wall approaches was primarily driven by behavior during the first night in the Social Box (Figure S1), suggesting that it may reflect altered responses to environmental novelty rather than a stable behavioral change. Similarly, in adulthood, ELA_Veh showed a significantly reduced chase‐to‐being‐chased ratio compared to Ctrl_Veh (Figure 2B). In contrast, ELA_SAFit2 mice did not differ significantly from Ctrl_Veh in any of the measured behaviors at either stage (Figure 2B). These findings suggest that ELA leads to persistent deficits in social behavior, which are effectively mitigated by SAFit2 treatment (Figure 2). In addition, using alternative parameterizations with ELA_Vehicle as the reference level yielded equivalent statistical results (Figure S2).

Finally, to investigate the temporal dynamics of behavioral alterations, readouts from the active phases were segmented into 2‐hour intervals and compared across Ctrl_Veh, ELA_Veh, and ELA_SAFit2 conditions. For behavioral features that showed significant differences between ELA_Veh and Ctrl_Veh, these differences were consistently observed across all 2‐hour time windows during the active phases (Figure S1), indicating that the group‐specific behavioral patterns were stable and persistent throughout the active period. These results underscore the robustness of ELA‐induced social deficits and highlight SAFit2 as a promising intervention capable of restoring stable behavioral dynamics across developmental stages and time. Importantly, none of the individual color coefficients were significant predictors of rank (all *p* > 0.20), indicating that coat color did not influence dominance status (Figure S3). In addition, to ensure that normalization to time spent outside the nest did not bias our behavioral readouts, we quantified nightly time spent inside the nest for each animal during the Social Box assessments (Figure S4). This analysis revealed no systematic condition‐dependent differences in nest occupancy, supporting that our normalization strategy controls for differences in opportunity to express behaviors rather than reflecting altered sleep‐like or withdrawal states.

Furthermore, maternal behavior during the ELA window (P02–P09) was investigated in a separate cohort using longitudinal day and night video recordings combined with advanced pose‐estimation tracking of maternal behavior, in order to determine whether SAFit2 rescue effects could be due to altered maternal behavior (Figure S5A). Specifically, nest‐directed behaviors were quantified, including entries into the nesting area and cumulative time spent in the nest. As expected, and shown in previous literature [32, 33], ELA robustly altered maternal nest‐related behaviors, reflected by changes in nest entries (Figure S5B) and time spent in the nest area (Figure S5C), but these ELA‐induced effects were not modified by SAFit2 treatment. These results indicate that the preventive effects of SAFit2 observed in the offspring are not driven by alterations in maternal behavior.

### 2.3 ELA‐Induced Social Subordination is Abolished by FKBP51 Inhibition via SAFit2 Treatment

**FIGURE 3:**
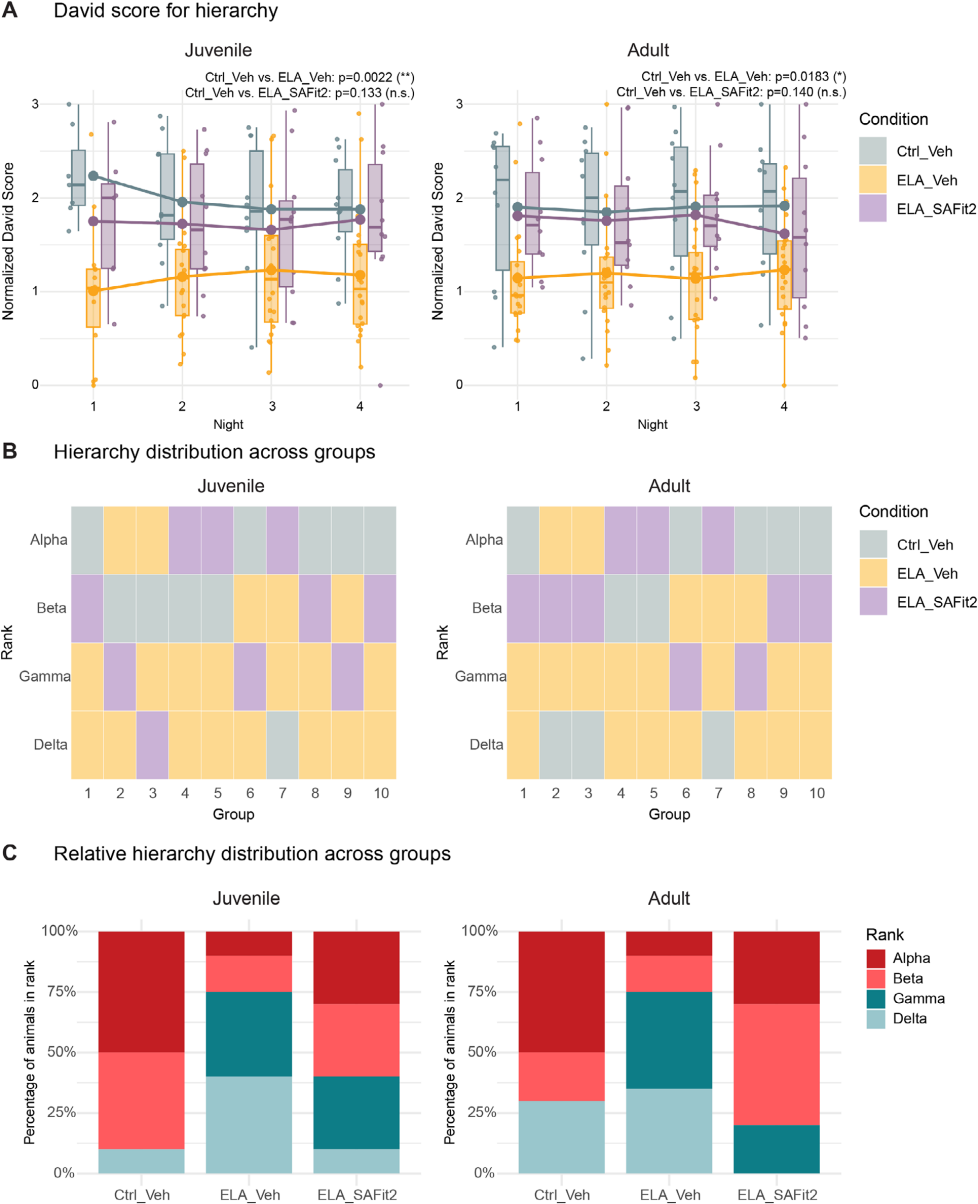
SAFit2 normalized ELA‐induced social subordination. (A) David's score of juvenile and adult animals. ELA_Veh mice showed significantly lower scores compared to Ctrl_Veh (*juvenile: F (1,95) = 9.91, p = 0.002; adult: F (1,104) = 5.74, p = 0.018*) while ELA_SAFit2 showed no significant difference (*juvenile: F (1,58) = 2.32, p = 0.133; adult: F (1,64) = 2.24, p = 0.140*). Two‐way repeated‐measures ANOVA. (B) Hierarchy distribution at juvenile and adult stages based on the cumulative David's score over 4 days. The hierarchy order from the highest ranking to lowest is alpha, beta, gamma, delta. ELA_Veh showed significantly different rank distribution compared to Ctrl_Veh (*juvenile: 𝜒2 (3, N = 30) = 11.86, p = 0.008; adult: 𝜒2 (3, N = 30) = 8.72, p = 0.033*), while ELA_SAFit2 showed no significant differences (*juvenile: 𝜒2 (3, N = 20) = 3.64, p = 0.303; adult: 𝜒2 (3, N = 20) = 6.79, p = 0.079*). Yate's corrected chi‐square test. (C) Relative hierarchy distribution within Ctrl_Veh, ELA_Veh, and ELA_SAFit2 at juvenile and adult stages. ELA_Veh has a lower percentage of dominant ranks (alpha, beta) than Ctrl_Veh (*juvenile: p = 0.001, odds ratio = 23.56, 95% CI [2.35, 1248.37]; adult: p = 0.045, odds ratio = 6.48, 95% CI [1.01, 55.16]*), while ELA_SAFit2 does not differ from Ctrl_Veh (*juvenile: p = 0.303, odds ratio = 5.49, 95% CI [0.41, 326.83]; adult: p = 1.00, odds ratio = 0.60, 95% CI [0.04, 6.94]*.). Fisher's exact test. Sample size: n = 10 for Ctrl_veh, n = 20 for ELA_Veh, n = 10 for ELA_SAFit2.

Social hierarchy provides a powerful representation of the social dynamics in group‐housed mice. To assess the impact of ELA and SAFit2 treatment on social dominance, we applied the well‐established David's score method [34, 35]. David's score is a widely used metric for quantifying social dominance based on agonistic interactions. It calculates the proportion of wins (chasing) and losses (being chased) relative to the total number of such interactions within a group, providing a continuous measure of individual dominance status derived from observed social behavior. At both adolescent and adult stages, ELA_Veh mice exhibited significantly lower David's scores compared to Ctrl_Veh mice, while ELA_SAFit2 animals did not differ significantly from controls (Figure 3A). This pattern was consistently observed across all four days of behavioral assessment (Figure 3A). The distribution of social ranks within each group also revealed significant differences across conditions (Figure 3B,C). ELA_Veh mice were disproportionately represented in lower social ranks (gamma and delta) relative to Ctrl_Veh and ELA_SAFit2 animals (Figure 3B). Conversely, fewer ELA_Veh mice occupied higher social ranks (alpha and beta), whereas ELA_SAFit2 mice showed a greater proportion in dominant positions, comparable to the rank distribution of Ctrl_Veh mice (Figure 3C). Together, these findings not only reinforce the link between early life adversity and social subordination, but also demonstrate that FKBP51 inhibition via SAFit2 treatment can effectively restore normative social hierarchy dynamics, offering a promising avenue for therapeutic intervention.

### 2.4 SAFit2 Restores ELA‐Disrupted Transcriptional Changes in Multiple Stress‐Related Brain Regions

To investigate how ELA and the pharmacological modulation via SAFit2 shape long‐term gene expression, we performed bulk RNA sequencing on a separate cohort with samples from six brain regions of adult males from the three conditions: Ctrl_Veh, ELA_Veh, and ELA_SAFit2 (n = 7 per condition). The selected regions are key areas in neural circuits regulating stress, emotion, and social behavior. Specifically, the medial prefrontal cortex (mPFC) regulates top‐down control of stress responses and social interactions, the anterior cingulate cortex (ACC) is central to emotional evaluation and stress reactivity, the dorsomedial thalamus (DMT) serves as an integrative hub linking prefrontal and limbic structures, the nucleus accumbens (Nacc) mediates motivation and reward sensitivity under stress, the basolateral amygdala (BLA) is critical for emotional memory and fear learning, and the ventral hippocampus (vHipp) contributes to stress regulation and contextual processing.

**FIGURE 4:**
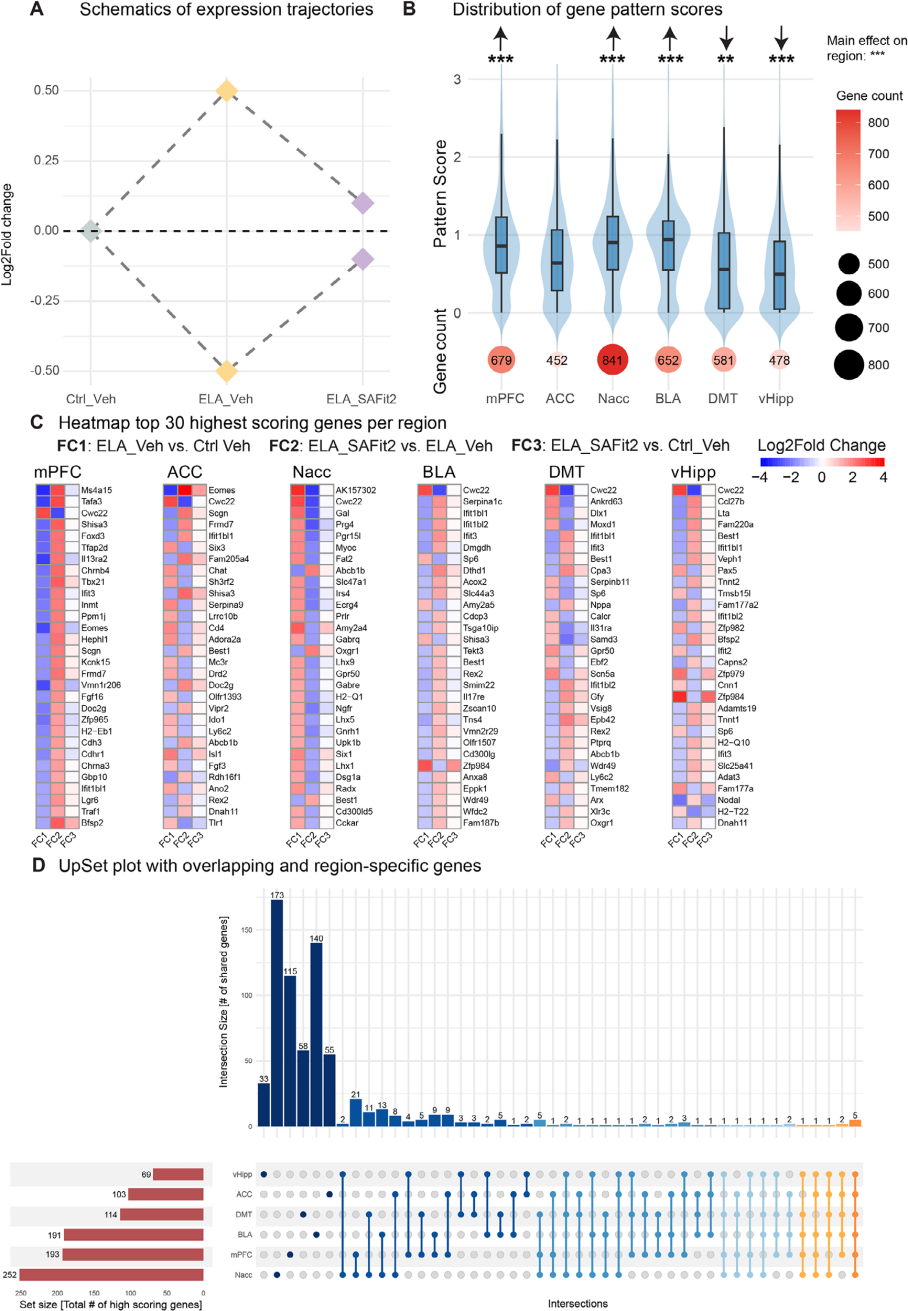
Distinct and region‐specific regulation patterns by ELA and SAFit2. (A). Schematic illustration of the expression trajectories following ELA and SAFit2 treatment identified using the pattern scoring approach. (B) Distribution of gene pattern scores and the number of dysregulated genes (*|Log2FC| > 0.5*) across six brain regions: medial prefrontal cortex (mPFC), anterior cingulate cortex (ACC), nucleus accumbens (Nacc), basolateral amygdala (BLA) dorsomedial thalamus (DMT), and ventral hippocampus (vHipp). The pattern score distribution differs significantly across regions (Kruskal‐Wallis test, *χ^2^(5) = 220.02, p < 2.2 × 10^−^^1^ ^6^*), with the arrows indicating the direction of the observed effects. Post hoc pairwise comparisons (Wilcoxon rank‐sum tests with FDR correction) revealed that pattern scores in the mPFC, NAcc, and BLA were comparable to each other (*mPFC vs. Nacc: p = 0.29, mPFC vs. BLA: p = 0.69, Nacc vs. BLA: p = 0.53*), but were significantly higher than those in DMT and vHipp (*mPFC vs. DMT: p = 6.9 × 10^−^^1^ ^6^, mPFC vs. vHipp: p < 2 × 10^−^^1^ ^6^, Nacc vs. DMT: p < 2 × 10^−^^1^ ^6^, Nacc vs. vHipp: p < 2 × 10^−^^1^ ^6^, BLA vs. DMT: p = 1.9 × 10^−^^15^, BLA vs. vHipp: p < 2 × 10^−^^1^ ^6^*). In contrast, DMT and vHipp did not differ significantly from each other (*DMT vs. vHipp: p = 0.19*), indicating a separation between regions with higher and lower pattern scores. The ACC showed intermediate values, differing significantly from all other regions (*ACC vs. mPFC: p = 4 × 10^−7^, ACC vs. Nacc: p = 5 × 10^−10^, ACC vs. BLA: p = 2.8 × 10^−7^, ACC vs. DMT: p = 0.01, ACC vs. vHipp: p = 7.3 × 10^−5^*). (C) Heatmap visualization of the log2 fold change of the top 30 highest scoring genes in each region. The three columns in each heatmap represent the three differential expression comparisons: FC1 = ELA_Veh vs. Ctrl Veh, FC2 = ELA_SAFit2 vs. ELA_Veh, FC3 = ELA_SAFit2 vs. Ctrl_Veh. The heatmaps are sorted by the highest to the lowest pattern scores from top to bottom. (D) UpSet plot showing the number of overlapping and region‐specific genes with high pattern score (>1.15, corresponding to the top 25th percentile) across the six regions. Sample size: n = 7 for Ctrl_veh, n = 7 for ELA_Veh, n = 7 for ELA_SAFit2.

We performed differential gene expression analysis with pairwise comparisons between Ctrl_Veh, ELA_Veh, and ELA_SAFit2. Only a limited number of genes were identified as differentially expressed with statistical significance (adjusted *p*<0.05), likely reflecting the subtle and regionally dispersed transcriptional changes that emerged in adulthood following ELA and SAFit2 treatment. Nevertheless, certain brain regions exhibited more pronounced shifts, with genes showing consistent fold‐change trends and lower p‐values. To further explore the transcriptomic data and capture biologically meaningful regulation, we filtered for genes with absolute Log2 fold change greater than 0.5 in at least one pairwise comparison, capturing those with robust and condition‐specific expression changes. The analysis also revealed distinct sets of genes whose expression was altered by ELA and, in some cases, restored by SAFit2 treatment. To systematically identify such gene expression trajectories, we devised and applied a pattern scoring approach to detect genes exhibiting changes consistent with ELA‐induced dysregulation and SAFit2‐mediated rescue. A higher pattern score indicates a greater likelihood that a gene follows this regulatory profile (Figure 4A). Pattern scores were calculated for all genes meeting the fold‐change threshold across the six regions. When comparing across brain regions, we observed variation in both the number of dysregulated genes and the distribution of pattern scores (Figure 4B). Notably, the mPFC, Nacc, and BLA showed more dynamic transcriptomic responses and a higher degree of gene patterning, while the remaining regions exhibited more modest shifts (Figure 4B). We also identified region‐specific regulation patterns among the top‐scoring genes (Figure 4C). In the mPFC, the top 30 highest scoring genes were predominantly downregulated by ELA and upregulated by SAFit2 treatment, whereas in the Nacc, the opposite pattern was observed, with an upregulation by ELA and downregulation by SAFit2 treatment (Figure 4C).

**FIGURE 5:**
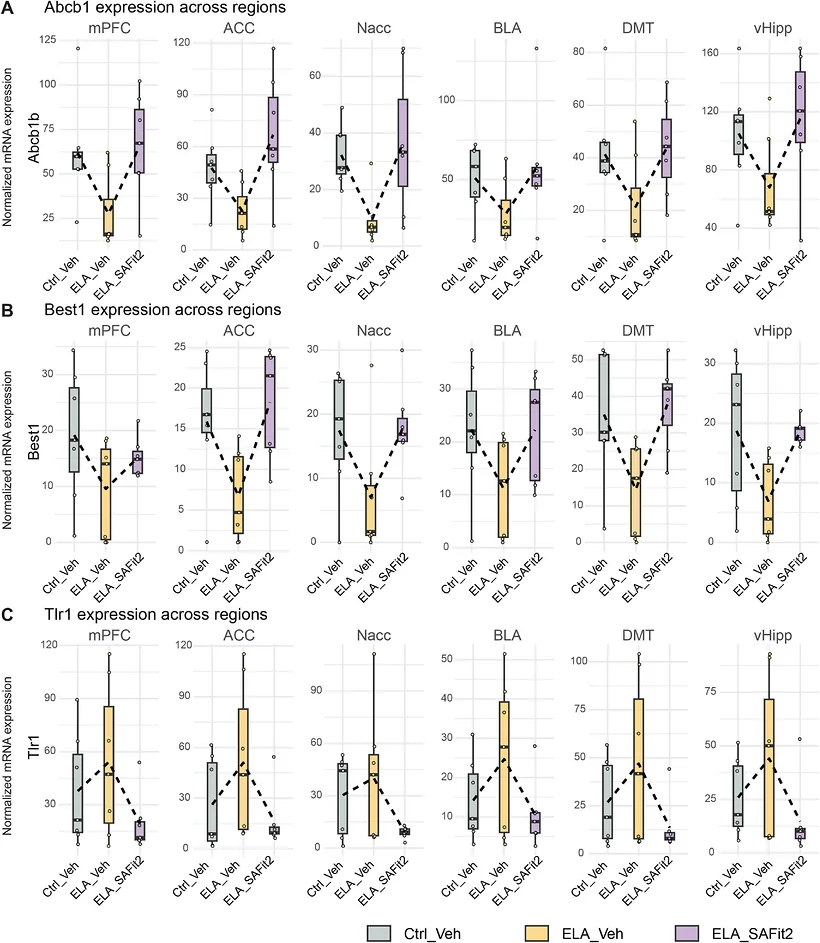
Genes of interest show consistent ELA‐SAFit2 expression trajectories across brain regions. (A) Consistent ELA‐induced downregulation restored by SAFit2 of *Abcb1b* in six brain regions. (Pattern scores—mPFC: 2.34, ACC: 2.13, Nacc: 3.55, BLA: 1.71, DMT: 1.89, vHipp: 1.32). (B) Consistent ELA‐induced downregulation restored by SAFit2 of *Best1* in six brain regions. (Pattern scores—mPFC: 1.29, ACC: 2.43, Nacc: 2.60, BLA: 1.96, DMT: 2.55, vHipp: 2.80). (C) Consistent ELA‐induced upregulation restored by SAFit2 of *Tlr1* in five brain regions. (Pattern scores—mPFC: 1.16, ACC: 1.93, Nacc: 0.80, BLA: 1.61, DMT: 1.63, vHipp: 1.53). A high pattern score (>1.15, corresponding to the top 25th percentile) suggests significant patterning following the expression trajectories. Expression across genes is displayed in normalized mRNA expression values. Sample size: n = 7 for Ctrl_veh, n = 7 for ELA_Veh, n = 7 for ELA_SAFit2.

To examine the convergence and divergence of genes with high pattern scores, we identified both region‐specific and cross‐regional gene sets (Figure 4D). While most genes displaying the pattern were unique to individual regions, a subset of genes followed the ELA‐SAFit2 regulation trajectory across multiple brain areas (Figure 4D and Table S3). Among this subset, *Abcb1b*, a transporter gene implicated in blood‐brain barrier regulation and stress responsivity, was downregulated by ELA and restored by SAFit2 (Figure 5A). Similarly, *Best1*, associated with astrocytic signaling and synaptic plasticity, showed reduced expression in ELA_Veh, which was restored in the ELA_SAFit2 condition (Figure 5B). Finally, the expression of *Tlr1*, a key component of the innate immune signaling pathway, was upregulated by ELA in five out of six regions, and attenuated by SAFit2 treatment (Figure 5C). These consistent expression trajectories across multiple regions suggest a shared molecular mechanism underlying ELA‐induced vulnerability and its reversal through FKBP51 inhibition by SAFit2 treatment.

### 2.5 ELA‐SAFit2‐Regulated Genes in mPFC and Nacc Reveal Distinct Functional Enrichment in Immune and Neuroactive Signaling Pathways

To uncover the biological significance of genes following the ELA‐SAFit2 regulation trajectory, we performed functional clustering using the Metascape gene annotation and functional enrichment tool [36], focusing on two brain regions with the most pronounced transcriptional patterning, the medial prefrontal cortex (mPFC) and nucleus accumbens (Nacc) (Figure S6). These regions were selected based on their distinct gene expression responses to ELA and SAFit2 treatment, as well as their central roles in stress regulation and social behavior. Genes with elevated pattern scores, indicating ELA‐induced dysregulation and SAFit2‐mediated rescue, were categorized into three groups: mPFC‐specific, Nacc‐specific, and overlapping (present in both regions). Functional clusters were then identified and visualized as network maps to reveal shared and region‐specific biological pathways (Figure S6A). Genes overlapping between mPFC and Nacc were significantly enriched in immunoregulatory interactions, suggesting a common inflammatory or immune‐related mechanism underlying ELA‐induced vulnerability and its reversal. In contrast, Nacc‐specific genes were predominantly associated with neuroactive ligand‐receptor signaling, pointing to regionally distinct modulation of neurotransmission and reward‐related processes (Figure S6B). These findings highlight that while SAFit2 exerts a broad rescuing effect on ELA‐induced transcriptional changes, the underlying biological pathways are regionally specialized. The convergence of immune‐related signaling in both regions, alongside divergent neurotransmission‐related pathways in the Nacc, underscores the complexity of stress‐related molecular adaptations and the precision of FKBP51‐targeted interventions. Together, these results suggest that SAFit2 not only abolishes ELA‐induced gene expression changes but also restores region‐specific molecular functions critical for emotional regulation and social behavior.

## 3 Discussion

In this study, we investigated if the pharmacological inhibition of the psychiatric risk factor FKBP51 can abolish the long‐term behavioral and brain‐wide transcriptional consequences of early life adversity. Our findings provide new evidence that targeting FKBP51 during the adversity period is a viable therapeutic strategy. Using an automated, and semi‐naturalistic phenotyping system, we demonstrated that ELA‐induced persistent deficits in social behavior are fully restored by early‐life treatment with the selective FKBP51 inhibitor SAFit2. To unravel the underlying molecular pathways related to this rescue, we performed transcriptional profiling across six stress‐relevant brain regions and revealed that SAFit2 effectively normalizes ELA‐induced gene expression changes, with the most pronounced effects seen in the mPFC and Nacc. Functional analysis of the rescued genes points to the reversal of dysregulated immune signaling and neuroactive ligand‐receptor pathways, suggesting that FKBP51 inhibition acts on core molecular processes that underpin the embedding of early adversity. Collectively, these results establish FKBP51 as a critical pharmacological target for reversing the lasting impact of early life adversity on social behavior and brain function.

Our findings underscore previous observations, indicating that exposure to early adversity leads to a deficit in social behavior, manifested as social subordination and reduced social engagement [19, 37]. This aligns with the clinical observations of social withdrawal and impaired social functioning in individuals with a history of childhood trauma [2, 38]. Crucially, we demonstrate that early‐life treatment with the selective FKBP51 inhibitor SAFit2 completely restored the normative social behavioral phenotype in male mice. To understand the mechanism underlying this behavioral rescue, we examined the physiological hallmarks of ELA. While SAFit2 treatment did not prevent the immediate physiological stress response to the limited bedding and nesting paradigm, it did promote a faster return to baseline body weight by P25 and likely moderated the embedding of persistent dysregulation of the HPA axis. The endocrine readouts revealed only partial alignment between pituitary and adrenal measures. At P09, SAFit2 treatment normalized ACTH levels in ELA animals, whereas corticosterone levels were modestly elevated. In adulthood, both ELA groups exhibited increased basal corticosterone levels despite the behavioral rescue observed in the SAFit2 treated group. This suggests that early FKBP51 inhibition preferentially impacts central regulation and feedback sensitivity of the HPA axis, while leaving aspects of adrenal output or maturation less amenable to long‐term normalization. A key function of FKBP51 is its role as a co‐chaperone of the GR‐complex, where it limits GR‐mediated negative feedback. We propose that strengthening glucocorticoid feedback during this critical period mitigates maladaptive central stress encoding and promotes long‐term behavioral resilience. The persistence of elevated corticosterone levels in adulthood, despite behavioral normalization, may reflect early‐life programming of adrenal sensitivity or accelerated HPA‐axis maturation induced by ELA, processes that may not be fully reversible by transient postnatal intervention [39, 40]. While peripheral mechanisms cannot be excluded, the brain penetrance of SAFit2 [22, 41], and the normalization of stress‐related transcriptional signatures support engagement of central FKBP51‐dependent pathways.

To uncover the molecular mechanisms underlying SAFit2‐mediated rescue, we applied a pattern scoring approach designed to identify genes that are dysregulated by ELA and subsequently restored by FKBP51 inhibition. This analysis revealed a regionally diverse transcriptional landscape, with the mPFC, NAcc, and BLA exhibiting the most prominent gene expression patterning in response to ELA and its pharmacological reversal. From a biological perspective, regions showing stronger transcriptional responses may be more directly engaged in processing stress‐related or social stimuli or may exhibit greater molecular plasticity, whereas regions with fewer detected changes may display subtler, coordinated transcriptional responses. Notably, a few genes showed consistent dysregulation across regions, whereas others followed region‐specific trajectories, highlighting both shared and distinct molecular responses to ELA and its reversal by SAFit2. Among the consistently dysregulated genes was *Abcb1b*, also known as multidrug resistance protein 1 (MDR1) or P‐glycoprotein 1 (P‐gp), a well‐characterized, ATP‐dependent efflux pump. Importantly, we previously identified *Abcb1b* as a regulator of glucocorticoid secretion in the adrenal cortex, upregulated in both chronically stressed mice, and human patients with Cushing's syndrome [42]. Our current findings suggest a potential role for *Abcb1b* in the brain, indicating that it may act as a peripheral‐central integrator of glucocorticoid dynamics. Supporting this, a common ABCB1 polymorphism (rs2032582) was also associated with a blunted cortisol response after corticotropin‐releasing factor stimulation in depressed human patients. These findings align with our preclinical data and support a conserved role for ABCB1/*Abcb1b* in regulating HPA axis dynamics across species. In addition, *Tlr1*, a Toll‐like receptor, has been implicated in neuroimmune signaling and psychiatric disorders [43], also showed consistent regulation across regions. Enrichment analyses most consistently highlighted immune‐related and neuroactive ligand‐receptor signaling pathways across regions; other functional categories were also detected but showed less cross‐regional consistency, and are therefore not emphasized here. This supports the idea that innate immune pathways are integral to the transcriptional response to ELA and its reversal by FKBP51 inhibition. However, given the sample size and the exploratory nature of the transcriptomics analysis, these findings should be interpreted as descriptive patterns rather than definitive effects. In this context, the highlighted genes may not act independently but instead represent functionally linked nodes within a broader stress‐immune regulatory network. Taken together, our findings highlight candidate pathways that could be explored in future studies to better understand how modulation of FKBP51 signaling propagates across endocrine and immune systems.

These results also build on our previous findings [19], which showed that ELA induces distinct transcriptional signatures in GABAergic and glutamatergic neurons of the ventral hippocampus. Using a comparable pattern scoring framework, we identified transcriptional motifs (i.e., chronic, primed, inverted, and blunted) that varied depending on ELA exposure and a second hit by acute stress, revealing nuanced gene regulation dynamics. In the present study, we did not observe a similar pronounced signal in the ventral hippocampus at the bulk tissue level. This discrepancy is likely due to methodological differences: prior findings were derived from single‐cell RNA sequencing, which enables cell‐type‐specific resolution of glutamatergic and GABAergic populations, whereas bulk RNA sequencing may mask opposing or cell type specific transcriptional changes. Thus, subtle excitation‐inhibition‐related alterations may not be detectable at the regional level in the current dataset. Nevertheless, our current bulk RNA‐seq findings complement the results from the previous study by providing a multi‐region, systems‐level perspective, suggesting that such transcriptional patterning may extend beyond the vHPC and reflect a broader, regionally distributed molecular signature of ELA that is responsive to FKBP51 inhibition. Importantly, the convergence of gene patterning across regions, particularly within immune and neuroactive ligand‐receptor pathways, parallels the functional clusters reported by Kos et al. [19]. Together, these findings suggest that FKBP51 inhibition with SAFit2 not only rescues behavioral impairments but also re‐establishes key molecular programs disrupted by early adversity. Though these conclusions should be interpreted within the constraints of the regions and resolution analyzed here, this convergence reinforces FKBP51's role as a key regulator of the transcriptional embedding of ELA and a promising target for its reversal. A methodological consideration is that the current design did not include a Ctrl_SAFit2 group, and therefore does not constitute a full 2 × 2 factorial manipulation of stress exposure and drug treatment. Instead, SAFit2 was deliberately deployed as a stress‑contingent intervention, with the 1:2:1 Social Box composition (Ctrl_Veh:ELA_Veh:ELA_SAFit2) optimized to maintain robust expression of ELA‑induced social impairments at the group level while allowing internal comparison to non‑stressed and rescued animals. This ethologically grounded group structure, together with continuous, high‑dimensional behavioral tracking, provides high sensitivity to detect how ELA shapes social dynamics and hierarchy formation, and how FKBP51 inhibition modulates these processes in a balanced social context. At the same time, the absence of a Ctrl_SAFit2 group means that ELA‑independent effects of early‑life FKBP51 inhibition in control animals cannot be excluded. Moreover, because animals from different conditions were co‑housed from weaning onward, stable dominance relationships likely emerged and persisted within these mixed‑condition groups, meaning that treatment effects and long‑term social dynamics are inherently intertwined and cannot be fully disentangled within the present design. Future studies using condition‑homogeneous housing (e.g., groups composed entirely of Ctrl_Veh, ELA_Veh, or ELA_SAFit2 mice) will be important to isolate intrinsic treatment effects from the emergent influence of group‑dependent hierarchy formation. Furthermore, an important consideration is the well‑documented sex‐specific effects of Fkbp5‐ELA interactions [44, 45], as well as sex‐dependent differences in social behavior previously observed in the Social Box following chronic stress exposure [46]. Extending the present paradigm to female mice therefore represents a critical next step. In particular, future studies should combine Social Box phenotyping with SAFit2 treatment in females after ELA to delineate sex‐dependent differences in stress processing and FKBP51 modulation with greater resolution. Another key question is whether FKBP51 inhibition can also reverse, rather than only prevent, ELA‐induced alterations when administered after the adversity period. From a translational standpoint, testing SAFit2 treatment during adolescence or adulthood, once ELA‐induced behavioral and molecular changes are established, would complement our current prevention‐focused design. Future studies should therefore systematically compare early‐life vs. post‐ELA SAFit2 intervention to delineate the therapeutic window and reversibility of ELA‐embedded phenotypes.

Taken together, our data show that early‐life inhibition of FKBP51 with SAFit2 prevents the embedding of early life adversity into persistent social subordination and brain‐wide transcriptional dysregulation. The administration of SAFit2 during the early life adversity window restored social behavior and social hierarchy, while normalizing ELA‐driven gene expression changes across several stress‐related brain regions, with pronounced effects in mPFC and Nacc. The rescued transcriptomic signatures converged on immunoregulatory processes and neuroactive ligand–receptor signaling, consistent with a SAFit2‐mediated strengthening of glucocorticoid feedback. Future work, including replications in independent cohorts and targeted functional studies, will be necessary to determine whether these molecular signatures play a causal role in the behavioral phenotypes observed. This positions FKBP51 as a critical pharmacological target for reversing the lasting impact of early life adversity on social behavior and brain function, offering a path toward developing targeted, preventative treatments for ELA‐related psychiatric disorders.

## 4 Materials and Methods

### 4.1 Animals and Housing Conditions

Wild‐type adult male and female CD1 mice (2‐3 months old) from the in‐house breeding facility of the Max Planck Institute of Psychiatry were used as breeders (F0). Their male offspring (F1) served as experimental animals. At weaning, F1 mice were housed in groups of four, with each cage containing animals from different litters, depending on the condition (see experimental design section). Mice were kept in individually ventilated cages (IVC; 30 × 16 × 16 cm, Tecniplast Green Line—GM500) connected to a central airflow system, under standard housing conditions: 12 h/12 h light–dark cycle (lights on at 7:00 a.m.), ambient temperature 23°C ± 1°C, and relative humidity 55%. Food (Altromin 1324, Altromin GmbH, Germany) and water were available ad libitum. See Table S1 to see the full lists of cohorts used across the different experiments. All experimental procedures were approved by the Committee for the Care and Use of Laboratory Animals of the Government of Upper Bavaria, Germany, and were conducted in accordance with the European Communities Council Directive 2010/63/EU.

### 4.2 Experimental Design

All breeding dams were primiparous and paired individually with one male, and dams were not co‐housed during mating. At postnatal day 2 (P02), litters were culled to a minimum of six and a maximum of ten pups to reduce variability in maternal care and nutritional load. Litters were then randomly assigned to control (Ctrl) or early life adversity (ELA) conditions, with dams receiving either vehicle (Veh) or SAFit2. At weaning (P25), offspring were housed according to a 1:2:1 design (Ctrl_Veh:ELA_Veh:ELA_SAFit2). The 1:2:1 composition was intentionally chosen to generate Social Box groups with an approximately balanced representation of animals expected to display ELA‐induced social impairments (2x ELA_Veh) vs. animals expected to be behaviorally unaffected (Ctrl_Veh) or rescued by treatment (ELA_SAFit2). Specifically, including two ELA_Veh animals per group increases the likelihood that ELA‑related behavioral alterations are expressed at the group level, while the inclusion of one Ctrl_Veh and one ELA_SAFit2 animal provides internal reference points for unaffected and pharmacologically rescued phenotypes. This results in groups composed of two affected and two unaffected animals, allowing robust assessment of social dynamics and hierarchy formation under conditions of mixed stress vulnerability.

### 4.3 The Early Life Adversity Paradigm: Limited Bedding and Nesting

Early life stress (ELA) was performed using the limited bedding and nesting (LBN) paradigm to induce chronic stress toward the mother and pups during P02 to P09, as previously described by Rice et al. [31]. At P02, all litters were transferred to new IVCs and randomly assigned to the non‐stressed (NS) or stressed (ELA) condition. If necessary, the litters were culled to a maximum of 10 animals per litter. The ELA litters were placed on a stainless‐steel mesh (McNichols) and were provided with limited nesting material (1/2 square of Nestlets, Indulab). The NS animals were placed in an IVC with a standard amount of bedding material and were provided with a sufficient amount of nesting material (2 squares of Nestlets). All litters were left undisturbed until P09, after which they returned to standard housing conditions. The pups were weaned in same‐sex groups with a maximum of four animals per cage.

### 4.4 Pharmacological Treatment With SAFit2

At P02, coinciding with the onset of the ELA paradigm, dams received a single subcutaneous injection of SAFit2 vesicular phospholipid lipid gel [47] (VPG; 200 µl, containing 10 mg SAFit2 per gram of gel). This formulation provides sustained release, resulting in stable plasma and brain concentrations with a half‐life of approximately 7 days [24, 48]. Pups were exposed to SAFit2 via maternal milk (Figure 1). Control animals received an injection of empty VPG without SAFit2 (Veh).

### 4.5 The Measurement of Adrenals and Corticosterone at P09 and Adulthood

One week after the behavioral tests, adult animals were weighed and sacrificed by decapitation. Trunk blood was collected in EDTA‐coated microcentrifuge tubes (Kabe Labortechnik, Germany) and immediately placed on ice. Samples were centrifuged at 4°C for 15 min at 8 000 rpm, after which plasma was separated and stored at −80°C until further analysis. Animals were sacrificed between 9:00–12:00, and the order of sacrifice was randomized across conditions.

A separate cohort of mice was used to obtain adrenocorticotropic hormone (ACTH) and corticosterone (CORT) measurements directly after ELA at P09. On the morning of P09, litters were kept in their home cage. The dam was sacrificed first, followed by the pups, which were kept in the nest until sacrifice to minimize additional acute stress. Trunk blood was collected and processed as described for adult samples. Plasma ACTH and CORT levels were measured in duplicate using radioimmunoassay, following the manufacturer's protocol (MP Biomedicals, Eschwege, Germany). Animals were sacrificed between 9:00–12:00, and the order of sacrifice was randomized across conditions.

Adrenal glands were dissected at P09 and P70 (adulthood) and kept in 0.9% NaCl at 4°C until further processing. Adrenals were processed in a randomized order within 3 days of sacrifice, during which surrounding fat tissue was removed and final adrenal weight was measured. Relative adrenal weight was calculated as the ratio of combined adrenal weight (left and right) to body weight measured prior to sacrifice.

### 4.6 Measurement of SAFit2 Concentration

In order to validate the effective transfer of SAFit2 via maternal milk and its subsequent systemic absorption, SAFit2 levels were measured in pup stomach content to provide a readout of ingested compound, while concurrent plasma measurements confirmed that this exposure translated into measurable circulating levels. Mice blood plasma samples were analysed using the combined high‐performance liquid chromatography/mass spectrometry (HPLC/MS‐MS) technique. Analysis was performed using a Shimadzu Nexera X2 (Shimadzu, Duisburg, Germany) liquid chromatograph which was interfaced to the ESI source of a Sciex QTrap 5500 (Sciex, Darmstadt, Germany) triple quadrupole mass spectrometer. All samples were prepared using Ostro protein precipitation and phospholipid removal plates (Waters, Eschborn, Germany).

Chromatography was accomplished using a gradient elution in an Accucore RP‐MS column (100×2, 1 mm, 2,6 µm Thermo Scientific, Dreieich, Germany) at a flow rate of 0.5 mL/min and 30°C. The composition of eluent B was methanol with 10 mm ammonium formate with 0,1% formic acid and water with 10 mm ammonium formate with 0,1% formic acid as eluent A. The gradient was 0–3 min 60% B, 3–4,5 min 60%–90% B, 0,5 min held at 90% B, 5‐5,2 min 90%–60% B and 5,2‐6 min 60% B. The total run time was 6 min and the injection volume was 2 µl. The ion source was operated in the positive mode at 500°C, and multiple reaction monitoring (MRM) collision‐induced dissociation (CID) were performed using nitrogen gas as the collision gas. Deuterated SAFit2 (SAFit2‐D3) was used as an internal standard. The retention time and transitions monitored during analysis for the analytes were as shown in the following table (Table 1).

**TABLE 1:** SAFit2 Analyte measurement standards.

Compound	Used as	Q1_Mass	Q3_Mass	RT [min]	DP [V]	EP [V]	CE [V]	CXP [V]
SAFit2	Quantifier	803,2	384,2	4,95	141	10	41	18
SAFit2	Qualifier	803,2	114,2	4,95	141	10	67	8
SAFit2‐D3	Internal Standard	806,4	384,3	4,95	106	10	41	26

### 4.7 Social Box (SB) Behavioral Assessment

Groups of male non‐sibling CD1 mice, consisting of one Ctrl_Veh, two ELA_Veh, and one ELA_SAFit2 animal, were housed together from weaning. Across the 10 Social Box groups (4 males per group), Ctrl_Veh, ELA_Veh, and ELA_SAFit2 animals originated from 10, 7, and 11 distinct litters, respectively, ensuring that each condition was represented by multiple independent litters (Tables 2 and 3). The fur of the animals was painted in different colors to keep track of their identity. Behavioral assessment in SBs took place at postnatal day 30 and 61, corresponding to adolescence and young adulthood. The use of SB phenotyping systems was described in detail previously [35, 46]. Each group was housed in a 60 cm × 60 cm living environment with two feeders for water and food, an enclosed nest and a smaller open nest, an s‐wall, and two ramps. The arenas were illuminated with around 200 lux during the light phase (12 h) and 2 lux during the dark phase (12 h). For each assessment, recording of the animals was done continuously in four dark phases and three light phases with a color‐sensitive camera.

**TABLE 2:** Litter composition for the Social Box groups.

Group	Litter ID (1: CTR_veh, 2–3: ELA_Veh, 4: ELA_SAFit2)
1	31, 25, 7, 1
2	20, 7, 22, 14
3	8, 7, 22, 18
4	4, 22, 3, 6
5	29, 3, 35, 32
6	15, 35, 3, 33
7	21, 3, 34, 14
8	4, 34, 17, 18
9	15, 34, 17, 9
10	20, 34, 17, 6

**TABLE 3:** Number of different litters per condition for the Social Box groups.

Condition	Total different number of litters
CTR_Veh	10
ELA_Veh	7
ELA_SAFit2	11

A custom neural network was trained with Deeplabcut [49, 50] (version 2.3.5) to analyze the videos and track the positions of body parts for each animal across all video frames. The resulting pose‐estimation data were parsed into 43 ethologically relevant behavioral readouts using a combination of heuristic algorithms and supervised machine‐learning classifiers [51]. Behavioral features were defined based on spatial relationships, movement dynamics, and temporal criteria, and were grouped into the following two categories: 1. Environment interactions, including interaction with SB objects, eating, drinking, resting, and exploration; 2. Social interaction, including chasing and sniffing events between two mice. All behavioral readouts and feature definitions are provided in Table S2. For downstream analyses, behavioral data were binned into multiple time windows (1‐, 2‐, 4‐, 6‐, 12‐hour bins) to capture both short‐term and long‐term dynamics. The duration and event count of each behavior were normalized by the time each animal spent outside the nest.

### 4.8 Analysis of Behavior Readouts

The influence of ELA and SAFit2 treatment on the behavioral features were assessed using linear mixed‐effects models implemented in the “nlme” package [52] in R (version 4.4.0). The behavioral features comprised heterogeneous data types, including event counts, durations, and derived ratios. To enable a unified modeling framework across all features, values were log‐transformed to reduce skewness and approximate normality prior to modeling. Observations with zero values for a given feature were excluded from that feature‐specific analysis.

For each feature, we fitted a linear mixed‐effects model with conditions and time window (night in the SB) as fixed effects, and random intercepts for individuals nested within experimental groups to account for repeated measurements and shared group‐level variance. The model can be expressed as:

$$log10\left(Y\right)∼Condition+TimeWindow+\left(1|Individual/Group\right)$$

where Y denotes the normalized behavioral feature.

The control group (Ctrl_Veh) was used as the reference level, such that model coefficients represent differences between ELA_Veh or ELA_SAFit2 relative to Ctrl_Veh. Alternative parameterizations using ELA_Vehicle as the reference level were also evaluated and yielded equivalent statistical results (Figure S2).

To summarize and visualize effects across behavioral features, we extracted regression coefficients and p‐values for fixed effects from each model. False discovery rate (FDR) adjustment was applied to the p‐values to account for multiple testing across behavioral features. The analyses were stratified by smaller time windows for the normalized David's score and specific behaviors, and two‐way repeated‐measures ANOVA was performed to assess condition‐specific effects over time.

### 4.9 Maternal Behavioral Assessment

Animals were recorded using the same hardware as in the Social Box experiment; however, the arena was smaller to more closely match the home‐cage environment (plexiglass arena, 25 × 25 cm, length × width). Mother and pups were placed in the recorded home‐cage arena at P02, immediately after SAFit2 or empty VPG injection, and were assigned to either the Ctrl or ELA condition. Maternal behavior was analyzed continuously during day and night from the start of the P03 light phase (7:00 a.m., lights on) until the end of the dark phase on P08, yielding six consecutive days and nights within the ELA window.

Instance segmentation was performed to detect the dam, using the real‐time transformer‐based RF‐DETR Seg model to generate object masks (small variant, 384 × 384 input, 100 object queries), a transformer‑based instance segmentation architecture derived from DETR [53] and Deformable‑DETR [54]. The model was trained on a custom mouse‑segmentation dataset annotated in Roboflow, initialized from standard COCO‑style pretrained weights, with the best checkpoint selected based on total validation loss. During inference, frames were sampled every fifth frame (stride = 5; effective rate ≈ 5 frames per second (fps) from a 25 fps source), and a custom GPU‑batched preprocessing pipeline replaced the per‑frame Python loop, achieving approximately 5–6× higher throughput (∼538 fps). Output masks were validated against the reference path by mask‑Intersection over Union (IoU) parity (mean IoU ≥ 0.95, minimum ≥ 0.80). The detection confidence threshold was set to 0.35, and for each frame the binary mask and confidence were stored as COCO‑RLE in Parquet format.

This approach yielded highly stable detection of the maternal mask across all videos. Nesting areas were manually drawn for every 12‑hour recording, and visual checks were performed to ensure that the nest was not displaced during the session so that the region of interest (ROI) remained valid. Behaviors occurring inside vs. outside the ROI were classified based on a minimum of 30% of the maternal mask overlapping with the ROI for at least 5 s. Finally, nest‐directed behaviors were quantified, including entries into the nesting area (total number) and cumulative time spent in seconds in the nest.

### 4.10 Transcriptomics

The selected regions are key areas in neural circuits regulating stress, emotion, and social behavior. Specifically, the medial prefrontal cortex (mPFC) regulates top‐down control of stress responses and social interactions, the anterior cingulate cortex (ACC) is central to emotional evaluation and stress reactivity, the dorsomedial thalamus (DMT) serves as an integrative hub linking prefrontal and limbic structures, the nucleus accumbens (Nacc) mediates motivation and reward sensitivity under stress, the basolateral amygdala (BLA) is critical for emotional memory and fear learning, and the ventral hippocampus (vHipp) contributes to stress regulation and contextual processing. Together, these six regions form a core stress‐ and social behavior‐related circuit, and our bulk RNA‐seq design was intended as a brain‐wide screen across these key nodes.

Following SB phenotyping under basal conditions, animals were sacrificed and brains were rapidly removed, snap‐frozen in 2‐methylbutane cooled on dry ice, and stored at −80°C until further processing. Coronal sections (250 µm) were cut on a cryostat, and tissue punches were bilaterally collected and pooled from the following regions (anteroposterior coordinates relative to bregma): medial prefrontal cortex, infralimbic and prelimbic (mPFC; AP +2.10–+1.54 mm); anterior cingulate cortex (ACC; AP +2.10–+0.50 mm); nucleus accumbens shell (Nacc; AP +1.94–+0.98 mm); basolateral amygdala (BLA; AP −0.70–−1.91 mm); dorsomedial thalamus (DMT; AP −0.70−1.91 mm); and ventral hippocampus (vHipp; AP −2.92–−3.64 mm). Punches were stored at −80°C until RNA isolation. Total RNA was isolated from punches using the miRNeasy Mini Kit (QIAGEN, Venlo, NL; cat. no. 1038703) according to the manufacturer's instructions, using RNase‐free technique throughout.

After RNA isolation, RNA integrity number (RIN) was measured using the Agilent 2100 Bioanalyzer system and quantified by Qubit (Thermo Fisher Scientific). RNAs with a RIN value > 7 were selected for mRNA sequencing (poly‐A selected). The libraries were prepared using the Illumina Stranded mRNA Prep, Ligation kit (Illumina), following the kit's instructions. After a final QC, the libraries were sequenced in a paired‐end mode (2×100 bases) in the Novaseq6000 sequencer (Illumina) using an S4.200 flowcell with a depth of ≥30 million paired‐reads per sample.

### 4.11 Quality Control and Differential Expression Analysis

The raw counts matrix was processed in R (version 4.4.0). A multi‐step filtering strategy was applied for the differential expression and downstream analysis. Lowly expressed and non‐informative genes were filtered prior to analysis. Specifically, genes with low cumulative count (<100), genes expressed in less than 6 samples (smallest group size), as well as non‐protein‐coding genes (according to Ensembl gene type annotation) were removed. The raw counts of samples from the same brain region were grouped and filtered together, and the filtered counts for each brain region were used for the subsequent differential expression analyses.

Differential gene expression analysis was performed in R (version 4.4.0) using the “DESeq2” package [55]. Pairwise comparisons were made within the three conditions: ELA_Veh vs. Ctrl_Veh, ELA_SAFit2 vs. ELA_Veh and ELA_SAFit2 vs. Ctrl_Veh. To balance biological relevance and statistical robustness, genes with |log2FC| > 0.5 in ELA_Veh vs. Ctrl_veh or ELA_SAFit2 vs. ELA_Veh were extracted for further analysis. To control for low‐expression bias toward fold change, we applied an additional expression filter, retaining only genes with mean normalized expression >5 in at least one of the conditions.

### 4.12 Transcriptional Patterning Calculations

To identify genes whose expression is altered by ELA and modulated by SAFit2 treatment, we defined a gene patterning score based on the log2 fold changes across three pairwise comparisons:

F*C1 = log2FC ELA_Veh vs. Ctrl_Veh (effect of ELA)*
*FC2 = log2FC ELA_SAFit2 vs. ELA_Veh (effect of SAFit2)*
*FC3 = log2FC ELA_SAFit2 vs. Ctrl_Veh (net effect relative to baseline)*

The pattern score was calculated as:

$$pattern\;score=\left|FC1-FC3\right|+\left|FC2-FC3\right|-\left|FC1+FC2\right|$$

This score was designed to prioritize genes for which SAFit2 induces a directional shift opposing the effect of ELA. Specifically, a higher gene patterning score indicates that gene expression is significantly altered by ELA and shifted in the opposite direction by SAFit2 treatment, consistent with a reversal or modulation of the ELA‐induced effects.

The distributions of gene patterning scores were visualized and compared across different brain regions. The effect of brain regions was assessed with Kruskal‐Wallis test, followed by pairwise Wilcoxon rank‐sum tests with FDR correction. The log2FC of the top 30 highest scoring genes in each region were visualized as a heatmap using “ggplot2” in R (version 4.4.0).

### 4.13 Metascape Gene Network Analysis

The metascape gene annotation and functional enrichment tool [36] was used to analyze genes with high patterning scores (above the 25th percentile) from mPFC and Nacc. The genes were divided into three lists, which consist of genes showing patterning only in mPFC, genes showing patterning only in Nacc, and genes showing patterning in both. The three gene lists were uploaded using the “multiple gene list” setting and analyzed with the express analysis settings. The gene enrichment clusters were visualized together with the contribution of the three gene sets (mPFC specific, Nacc specific, overlapping) to each of the identified clusters.

### 4.14 Statistics

Statistical analyses and data visualization for the physiological hallmarks of early life stress (Figure 1) were performed using GraphPad Prism 10 and RStudio (R version 4.4.0). Outliers were identified with Grubbs’ test and excluded when significant. Assumptions of normality were evaluated with the Anderson‐Darling test. When assumptions were violated, non‐parametric alternatives were applied. Comparisons among three groups were conducted using one‐way ANOVA (parametric) or Kruskal‐Wallis test (non‐parametric). Significant main effects were followed by post hoc analyses using the Holm–Šidák test (parametric) or Dunn's test (non‐parametric). Bar graphs are presented as the mean ± standard deviation (SD). Data were considered significant at *p* < 0.05 (^*^), and further significance was represented as *p* < 0.01 (^**^), *p* < 0.001 (^***^), and *p* < 0.0001 (^****^).

## Author Contributions

**Conceptualization**: J.B., J.P.L., M.V.S; **Experimental design**: J.B., X.J., J.P.L., M.V.S.; **Data analysis**: J.B., X.J., C.N., A.K., C.B.; **Software**: X.J., S.G., C.S.C.; **Investigation**: J.B., X.J., D.H., C.F., C.N.; **Validation**: J.B., X.J.; **Resources**: M.U., G.W., F.H., A.C., J.P.L., M.V.S.; **Data Curation**: J.B., X.J., P.F., J.P.L., M.V.S.; **Visualization**: J.B., X.J.; **Supervision**: C.B., P.F., J.P.L., M.V.S.; **Writing – Original Draft**: J.B., X.J., J.P.L., M.V.S.; **Writing – Editing**: All authors; **Project Administration**: J.P.L., M.V.S.; **Funding Acquisition**: J.P.L., M.V.S.

## Funding

This study is supported by the “Kids2Health” grant of the Federal Ministry of Education and Research [01GL1743C]. JPL receives research funding from the Swedish Society for Medical Research (SSMF) (Grant No.: SG‐22‐0204), the Swedish Research Council (VR) (Grant No.: 2023‐02499), the Swedish Brain Foundation (Hjärnfonden) (Grant Nos.: FO2023‐0313 and FO2024‐0038), the Strategic Research Area Neuroscience (StratNeuro), and the European Research Council (ERC) (Grant No.: 101116064) through a Starting Grant.

## Conflicts of Interest

The authors declare no conflicts of interest.

## Supporting information




**Supporting File**: advs76040‐sup‐0001‐SuppMat.docx.

## Data Availability

The data that support the findings of this study are openly available in Gene Expression Omnibus at https://www.ncbi.nlm.nih.gov/geo/, reference number GSE318028.
