# Supplementary material for: Pharmacological Inhibition of FKBP51 Mitigates Early Life Adversity‐Induced Social Deficits in Male Mice
Source: Adv Sci (Weinh). 2026 Jun 9;13(48):e76040. doi: 10.1002/advs.76040 (PMC13336721; doi:10.1002/advs.76040)
Supplement: Supplementary file 1 [file ADVS-13-e76040-s001.docx]

**Supplementary Materials**

| **Figure** | **Cohort (CT)** |
| --- | --- |
| 1B | CT1 (p04), CT2 (p07), CT3 (P09) |
| 1C,D,E,I,J | CT4 |
| 1F | CT5,6 |
| 1G,H | CT5 |
| 2,3, Supplementary Figure 1, 2, 3, 4 | CT6 |
| 4,5, Supplementary Figure 6 | CT7 |
| Supplementary Figure 5 | CT8 |

**Supplementary table 1.** The different cohorts of animals used across the figures.

| **Category** | **Name** | **Definition** |
| --- | --- | --- |
| Social interaction | Sniffing vs. sniffed - Event count | The number of sniffing events initiated by the animal divided by the number of events where the animal is being sniffed by another animal. |
|  | Sniffing - Time | The total duration of the sniffing events initiated by the animal, normalized by the total time the animal spent outside the nest. |
|  | Sniffed - Time | The total duration of events in which the animal is being sniffed by another animal, normalized by the total time the animal spent outside the nest. |
|  | Sniffing - Event count | The number of sniffing events initiated by the animal, normalized by the total time the animal spent outside the nest. |
|  | Sniffed - Event count | The number of events in which the animal is being sniffed by another animal, normalized by the total time the animal spent outside the nest. |
|  | Head-to-head sniffing - Time | The total duration of head-to-head sniffing events (both animals oriented face-to-face), normalized by the total time the animal spent outside the nest. |
|  | Head-to-side sniffing - Time | The total duration of events in which the animal sniffs the side or flank of another animal, normalized by the total time the animal spent outside the nest. |
|  | Head-to-side sniffed - Time | The total duration of events in which the animal's side or flank is being sniffed by another animal, normalized by the total time the animal spent outside the nest. |
|  | Anogenital sniffing - Time | The total duration of events in which the animal sniffs the anogenital region of another animal, normalized by the total time the animal spent outside the nest. |
|  | Anogenital sniffed - Time | The total duration of events in which the animal's anogenital region is being sniffed by another animal, normalized by the total time the animal spent outside the nest. |
|  | Chasing vs. chased - Time | The total duration of chasing events initiated by the animal divided by the total duration of events in which the animal is being chased by another animal. |
|  | Chasing vs. chased - Event count | The number of chasing events initiated by the animal divided by the number of events in which the animal is being chased by another animal. |
|  | Chasing - Time | The total duration of chasing events initiated by the animal, normalized by the total time the animal spent outside the nest. |
|  | Chased - Time | The total duration of events in which the animal is being chased by another animal, normalized by the total time the animal spent outside the nest. |
|  | Chasing - Event count | The number of chasing events initiated by the animal, normalized by the total time the animal spent outside the nest. |
|  | Chased - Event count | The number of events in which the animal is being chased by another animal, normalized by the total time the animal spent outside the nest. |
| Environment interaction | Distant water - Time | The total duration the animal spent at the water source located farther from the nest (distant), normalized by the total time the animal spent outside the nest. |
|  | Distant water - Approaches | The number of approaches the animal made to the distant water source, normalized by the total time the animal spent outside the nest. |
|  | Proximal water - Time | The total duration the animal spent at the water source located closer to the nest (proximal), normalized by the total time the animal spent outside the nest. |
|  | Proximal water - Approaches | The number of approaches the animal made to the proximal water source, normalized by the total time the animal spent outside the nest. |
|  | Distant feeder - Time | The total duration the animal spent at the food source located farther from the nest (distant), normalized by the total time the animal spent outside the nest. |
|  | Distant feeder - Approaches | The number of approaches the animal made to the distant food source, normalized by the total time the animal spent outside the nest. |
|  | Proximal feeder - Time | The total duration the animal spent at the food source located closer to the nest (proximal), normalized by the total time the animal spent outside the nest. |
|  | Proximal feeder - Approaches | The number of approaches the animal made to the proximal food source, normalized by the total time the animal spent outside the nest. |
|  | Distant vs. proximal water - Time | The total duration the animal spent at the distant water source divided by the total duration spent at the proximal water source. |
|  | Distant vs. proximal water - Approaches | The number of approaches to the distant water source divided by the number of approaches to the proximal water source. |
|  | Distant vs. proximal feeder - Time | The total duration spent at the distant food source divided by the total duration spent at the proximal food source. |
|  | Distant vs. proximal feeder - Approaches | The number of approaches to the distant food source divided by the number of approaches to the proximal food source. |
|  | Feeder - Total time | The total duration the animal spent at any food source (proximal or distant combined), normalized by the total time the animal spent outside the nest. |
|  | Water - Total time | The total duration of time the animal spent at any water source (proximal or distant combined), normalized by the total time the animal spent outside the nest. |
|  | Center arena - Time | The total duration of time the animal spent in the center of the arena (away from the walls), normalized by the total time the animal spent outside the nest. |
|  | Center arena - Entries | The number of times the animal entered the center of the arena (away from the walls), normalized by the total time the animal spent outside the nest. |
|  | Ramp1 - Time | The total duration of time the animal spent on ramp 1, normalized by the total time the animal spent outside the nest. |
|  | Ramp1 - Approaches | The number of approaches the animal made to ramp 1, normalized by the total time the animal spent outside the nest. |
|  | Ramp2 - Time | The total duration of time the animal spent on ramp 2, normalized by the total time the animal spent outside the nest. |
|  | Ramp2 - Approaches | The number of approaches the animal made to ramp 2, normalized by the total time the animal spent outside the nest. |
|  | S-wall - Time | The total duration of time the animal spent at or near the S-shaped wall structure, normalized by the total time the animal spent outside the nest. |
|  | S-wall - Approaches | The number of approaches the animal made to the S-shaped wall structure, normalized by the total time the animal spent outside the nest. |
|  | Enrichment - Total time | The total duration of time the animal spent interacting with any enrichment object (s-wall and ramps) in the arena, normalized by the total time the animal spent outside the nest. |
|  | Small nest - Time | The total duration of time the animal spent inside the smaller, open nest area, normalized by the total time the animal spent outside the nest. |
|  | Small nest - Entries | The number of times the animal entered the small nest area, normalized by the total time the animal spent outside the nest. |
|  | Nest - Time | The proportion of time the animal spent inside the closed nest in the wholetime window (i.e., total nest time divided by total observation time). |
|  | Nest - Entries | The total number of times the animal entered the closed nest. |

**Supplementary Table 2.** List of behavioral readouts and definitions.

| Gene name | Description | Pattern score - mPFC | Pattern score - ACC | Pattern score - Nacc | Pattern score - BLA | Pattern score - DMT | Pattern score - vHipp |
| --- | --- | --- | --- | --- | --- | --- | --- |
| *Abcb1b* | ATP-binding cassette, sub-family B member 1B | **2.34** | **2.13** | **3.55** | **1.71** | **1.89** | **1.32** |
| *Best1* | Bestrophin 1 | **1.29** | **2.43** | **2.60** | **1.96** | **2.55** | **2.80** |
| *Cwc22* | CWC22 spliceosome-associated protein | **5.45** | **5.70** | **5.55** | **5.59** | **5.77** | **5.89** |
| *H2-T22* | Histocompatibility 2, T region locus 22 | **1.37** | 1.07 | **1.35** | **1.21** | **1.49** | **1.57** |
| *Ifit1bl1* | Interferon induced protein with tetratricopeptide repeats 1B like 1 | **2.72** | **3.18** | **1.49** | **3.06** | **2.98** | **2.73** |
| *Ifit2* | Interferon-induced protein with tetratricopeptide repeats 2 | **1.67** | **1.68** | **1.78** | **1.71** | 0.22 | **1.99** |
| *Ifit3* | Interferon-induced protein with tetratricopeptide repeats 3 | **3.93** | **1.81** | 0.84 | **2.83** | **2.67** | **1.62** |
| *Sp6* | Trans-acting transcription factor 6 | 0.66 | **1.22** | **1.27** | **2.34** | **2.30** | **1.65** |
| *Tlr1* | Toll-like receptor 1 | **1.16** | **1.93** | 0.80 | **1.61** | **1.63** | **1.53** |
| *Zfp984* | Zinc finger protein 984 | **1.28** | **1.24** | **1.49** | **1.81** | **1.42** | **1.74** |

**Supplementary Table 3.** Genes with high pattern scores across regions (Pattern score > 1.15 in five or more regions)

**Supplementary Figure 1.** **Time-resolved profiles of significant behavioral features. A-D).** Behavioral readouts from the active phases were segmented into 2-hour time windows and compared across Ctrl_Veh, ELA_Veh and ELA_SAFit2 conditions. **A).** Ratio between the time spent chasing and the time being chased. **B).** Ratio between the event count of chasing and being chased. **C).** The total time spent chasing. **D).** The event count of chasing. **E).** The number of approaches to the s-wall. The time and number of events are normalized by the total time the animal spent outside the nest.

**Supplementary Figure 2. Social Box Behaviors.** Linear mixed-effects model analyses of behavioral readouts, using ELA_Veh as reference group and comparing Ctrl_Veh vs. ELA_Veh and ELA_SAFit2 vs. ELA_Veh using a linear mixed-effect model. FDR adjustment was applied on p-values. Behavior readouts showing statistically significant difference are marked with *. Sample size: n=10 for Ctrl_veh, n=20 for ELA_Veh, n=10 for ELA_SAFit2.

**Supplementary Figure 3. Hierarchy rank out from the Social Box.** A) Distribution of hierarchy ranks in different coat colors. We assessed whether assigned color predicted social rank using ordinal logistic regression. No significant effect of color on rank was detected at either age examined (likelihood ratio test, P30*: χ²(3) = 2.12, p = 0.55;* 2 months: *χ²(3) = 6.11, p = 0.11*). Consistently, none of the individual color coefficients were significant predictors of rank (*all p > 0.20*), indicating that coat color did not influence dominance status. B) Distribution of experimental conditions in different coat colors. Color assignment was randomized across experimental conditions. Sample size: n=10 for Ctrl_Veh, n=20 for ELA_Veh, n=10 for ELA_SAFit2.

**Supplementary Figure 4.** **Time spent inside the nest during behavior assessments of the Social Box.** The left panel shows the behavior assessment during adolescence (postnatal day 30 to 34) and the right panel shows the behavior assessment during early adulthood (postnatal day 61 to 65). The individual data points are colored by the experimental condition and correspond to the total time each animal spent inside the nest in an active phase (12h) during the behavior assessments. Each assessment includes 4 active phases, 10 groups with 1 Ctrl_Veh, 2 ELA_Veh, and 1 ELA_SAFit2 animal in each group.

**Supplementary Figure 5.** **Longitudinal recordings of maternal behavior during ELA P-02-9. A).** Behavioral set-up and example region of interest of the nesting area in the ELA home-cage arena. **B).** Cumulative plot of the total time spent in the nest area. The area-under-the-curve (AUC) analysis revealed a significant main effect of condition in a one-way ANOVA (F(2,16) = 5.39, p = 0.0162). Follow-up Welch’s t tests indicated a significantly reduced AUC for Ctrl_Veh compared to ELA_Veh (p = 0.0424) and ELA_SAFit2 (p = 0.0206), whereas no difference was observed between ELA_Veh and ELA_SAFit2 (p = 0.6070). **C).** Cumulative plot of the total number of entries into the nest area. The AUC analysis revealed a significant main effect of condition in a one-way ANOVA (F(2,16) = 14.67, p = 0.00024). Follow-up Welch’s t tests indicated a significantly reduced AUC for Ctrl_Veh compared to ELA_Veh (p = 0.0133) and ELA_SAFit2 (p = 0.00111), whereas no difference was observed between ELA_Veh and ELA_SAFit2 (p = 0.0742). Timelines show the mean +/- 95% confidence interval, bar plots show the mean +/- standard deviation.

**Supplementary Figure 6.** **Functional clustering of genes with elevated pattern scores in the mPFC and Nacc.** **A).** Network map visualization of the identified functional clusters. The pathways are colored with mPFC-specific, Nacc-specific and overlapping (present in both regions). **B).** Clustered heatmap of the top 20 biological processes enriched in mPFC-specific, Nacc-specific and overlapping genes.
